# Features of COPD patients by comparing CAT with mMRC: a retrospective, cross-sectional study

**DOI:** 10.1038/npjpcrm.2015.63

**Published:** 2015-11-05

**Authors:** Wei-Chang Huang, Ming-Feng Wu, Hui-Chen Chen, Jeng-Yuan Hsu, The TOLD study group

**Affiliations:** 1 Division of Chest Medicine, Department of Internal Medicine, Taichung Veterans General Hospital, Taichung, Taiwan; 2 Department of Medical Technology, Jen-Teh Junior College of Medicine, Nursing and Management, Miaoli, Taiwan; 3 Department of Medical Laboratory Science and Biotechnology, Central Taiwan University of Science and Technology, Taichung, Taiwan; 4 Division of Critical Care and Respiratory Therapy, Department of Internal Medicine, Taichung Veterans General Hospital, Taichung, Taiwan; 5 School of Medicine, China Medical University, Taichung, Taiwan; 6 School of Physical Therapy, Chung-Shan Medical University, Taichung, Taiwan

## Abstract

**Background:**

The group assignment of chronic obstructive pulmonary disease (COPD) may differ depending on whether the COPD assessment test (CAT) or modified Medical Research Council dyspnoea scale (mMRC) is used.

**Aims:**

This study intended to clarify how different patient characteristics influence the differences, to determine the relationships between CAT and mMRC and to characterise COPD patients by both CAT and mMRC.

**Methods:**

This was a retrospective, cross-sectional study. The data, collected by Taiwan Obstructive Lung Disease consortium, were managed and analysed.

**Results:**

Of the 757 participants, COPD group assignment was not identical as well as no substantial agreement presented when categorised based on the cut-point CAT score ⩾10 and each mMRC cut-point. In all, 38.2% of participants had discordant group assignments together with a lower mean CAT score, less severe airway obstruction and less severe airflow limitation compared with those with concordant group assignments. In the discordant group, the CAT⩾10/mMRC 0–1 subgroup had more wheezing than CAT<10/mMRC⩾2 subgroup. Only moderate correlations existed between CAT and mMRC. More-symptom groups and combined high-risk group had better correlations than less-symptom groups and combined low-risk group, respectively. A modest negative correlation existed between forced expiratory volume in 1 s percentage (FEV_1_%) predicted and CAT score and between FEV_1_% predicted and mMRC scale in parallel with a significant positive relationship existing between the CAT score and mMRC scale. Notably, a significant proportion of COPD patients with each scale of mMRC had health status impairment.

**Conclusions:**

The Global initiative for Chronic Obstructive Lung Disease committee should redefine the applications of CAT and mMRC in the management of COPD.

## Introduction

The complexities of chronic obstructive pulmonary disease (COPD) require a comprehensive assessment for its management. The Global initiative for Chronic Obstructive Lung Disease (GOLD) committee has provided recommendations for the appropriate diagnosis and treatment of COPD. Emerging evidence indicates that the degree of airflow limitation was poorly predictive of dyspnoea and quality of life.^1–3^ Therefore, the GOLD committee in 2011 moved away from a linear, one-dimensional classification of severity groups, defined solely by degree of airflow limitation (forced expiratory volume in 1 s (FEV_1_)), to a two-dimensional assessment that takes into account both exacerbation risk and symptom assessment.^4^

The exacerbation risk of COPD is determined by exacerbation history in the previous 1 year and spirometric classification of airflow limitation by the GOLD grade categorised by FEV_1_% predicted. This symptom is measured by either the COPD assessment test (CAT) or the modified Medical Research Council dyspnoea scale (mMRC) although the Clinical COPD Questionnaire was also proposed by GOLD 2013 revision. High risk includes GOLD 3 or 4 (severe or very severe) or a history of ⩾2 exacerbations in the previous year. The highest risk should be used if there is a discrepancy between the risk category as determined by the spirometric classification and that derived by the exacerbation history. Otherwise, low risk will be the case. The COPD patient’s level of symptoms is classified into either less or more symptoms. A CAT score ⩾10 or mMRC scale ⩾2 indicates more symptoms, otherwise less symptoms will be the case. This combination of assessments classifies COPD patients into one of the four categories: A (low risk and less symptoms), B (low risk and more symptoms), C (high risk and less symptoms), and D (high risk and more symptoms). The management strategy is thereby determined according to this classification.

The CAT comprises eight items relating to the severity of cough, sputum, dyspnoea, chest tightness, capacity for exercise and activities, confidence, sleep quality and energy levels,^5^ while the mMRC is a quantitative assessment tool only for breathlessness.

Although GOLD 2011 recommended a CAT score ⩾10 corresponding to a mMRC scale ⩾2 in symptom assessment for categorising COPD patients into less- or more-symptom groups, several studies have found that the group assignment of COPD produced by these cut-points was not identical.^6–8^

To understand the impacts of GOLD 2011 on COPD patients in Taiwan, the Taiwan Obstructive Lung Disease (TOLD) consortium was set up and is comprised of 12 teaching hospitals throughout Taiwan. We hypothesised that group assignment of COPD by the cut-point CAT score ⩾10 was not exactly the same with that by the cut-point mMRC scale ⩾2. The aim of this study was to clarify how different patient characteristics influence the differences, to determine the relationships between CAT and mMRC and to characterise COPD patients by both CAT and mMRC, as implemented by the TOLD consortium.

## Materials and methods

### Study design and population

The data were from a large-scale, cross-sectional, multi-centre, observational, retrospective study of the TOLD consortium conducted between November 2012 and August 2013 in 12 teaching hospitals throughout Taiwan. The participating physicians, who were qualified pulmonologists and actively involved in COPD management, screened outpatients for study entry. Patients aged ⩾40 years with a confirmed diagnosis of COPD based on the GOLD 2011 recommendation and a spirometry within the previous 1 year before enrollment were invited to participate. Patients were excluded if they participated in interventional clinical trials in the previous 1 year, had a history of asthma or, for the purpose of this study, did not complete both the CAT and mMRC. The hospitals’ individual Institutional Review Board and Ethics Committees approved this study (approval number: CE13164) and informed consent was obtained from all participants.

### Data collection

At a single study visit, participating physicians completed a detailed patient record form, which included baseline characteristics, smoking history, presence of absence of wheezing on listening to the chest at the outpatient clinics in the previous 1 year, spirometry, CAT scores, mMRC scales, exacerbation history in the previous 1 year, comorbidities of interest, including cardiovascular diseases (e.g., ischaemic heart disease, congestive heart failure, hypertension and arrhythmia), chronic lung diseases (e.g., previous pulmonary tuberculosis, bronchiectasis and pneumoconiosis) and lung cancer, and COPD groups according to the GOLD 2011 recommendation for each participant from medical records. After that, the patient record forms were collected for further data management and analysis.

### Symptom assessment

The CAT is a questionnaire that is designed to measure health status of COPD patients. Eight statements assess the best and worst case scenarios of cough, phlegm, chest tightness, breathlessness going up hills/stairs, activity limitations at home, confidence leaving home, sleep and energy. Each statement is scored from 0 to 5 (best to worst) giving a total score range from 0 to 40.^5^ The mMRC, which is a patient-reported ordinal-rating scale, comprises five statements that describe almost the entire range of respiratory disability from none (Grade 0) to almost complete incapacity (Grade 4).^9^ For the purpose of this study, symptom evaluation for a GOLD grouping (A–D) was carried out using both questionnaires in each participant.

### Exacerbation risk

Spirometry was taken from the documented evidence within the previous 1 year and interpreted according to the American Thoracic Society statement.^10^ Positive bronchodilator test (BT) was defined as FEV_1_ or forced vital capacity (FVC) improvement from predose value by ⩾12% and ⩾200 ml. An acute exacerbation was defined as a worsening of symptoms that required antibiotics or systemic steroids, emergency room visits or hospitalisations. A history of ⩾2 exacerbation in the previous 1 year was termed as frequent exacerbation. A participant with frequent exacerbation was referred to as a frequent exacerbator; otherwise non-frequent exacerbator was the referent.

### COPD patient group

Participants were classified into four groups—A, B, C or D—by their COPD symptoms as determined by CAT or mMRC and exacerbation risks as determined by GOLD spirometric classification and the history of exacerbations in the preceding year according to the GOLD 2011.^4^ However, for the purpose of this study, each individual participant was assigned twice, one with a CAT score and the other with the mMRC scale.

### Statistical analysis

All data were expressed as mean and s.d. for continuous variables or number (percentage) for categorical variables. Comparisons were conducted using the independent *t*-test for continuous variables and chi-square test for categorical variables. Pearson’s correlation coefficient was applied to test the relationship between CAT scores and mMRC scales, between post-BT FEV_1_% predicted and the CAT score and between post-BT FEV_1_% predicted and the mMRC scale. Analysis of variance was applied to test the association between the CAT score and mMRC scale. The kappa coefficient was used to interpret the extent of agreement between the two respiratory questionnaires (CAT versus mMRC), where kappa<0 indicates a less than chance agreement and kappa=1 indicates a perfect agreement. Statistical significance was set at *P*<0.05. Statistical analysis was performed using the SPSS version 18.0 (SPSS, Chicago, IL, USA).

## Results

This observational study was conducted in 12 teaching hospitals throughout Taiwan, where 1054 subjects who came to outpatient clinics for any reason and who had a diagnosis of COPD were enrolled. However, out of the 1054 subjects, 297 subjects without the completion of both questionnaires were further excluded. In the end, 757 subjects who fulfilled the inclusion and exclusion criteria were included in the final analysis.

Table 1 shows the baseline characteristics of the enrolled participants. The overall mean age was 72.2±9.4 years and the majority of participants were male. Cigarette smoking was the leading cause of COPD in 92.1% (697/757) of participants. Interestingly, frequent exacerbation, wheezing and positive BT were present in 13.6, 44.8 and 30.5% of the enrolled subjects, respectively.

For the purpose of this study, we evaluated the GOLD groups twice for each participant, once using the CAT score and again using the mMRC scale. Table 2 indicates that COPD group assignment was not identical when categorised based on the cut-point CAT score ⩾10 and each mMRC cut-point. Based on the cut-points CAT score ⩾10 and mMRC scale ⩾2 recommended by the GOLD 2011, classifying patients by CAT resulted in 30.6, 17.3, 22.2 and 29.6% of participants in groups A, B, C and D, whereas by mMRC resulted in 22.2, 26.0, 1.9 and 35.9%, respectively. The agreement of group assignment was evaluated between by the cut-point CAT score ⩾10 and by using different cut-points of the mMRC scale. The best agreement of group assignment emerged when the cut-point CAT score ⩾10 corresponded to the cut-point mMRC ⩾3 (kappa=0.55, *P*=0.000), whereas the worst agreement emerged when the cut-point CAT score ⩾10 corresponded to the cut-point mMRC ⩾1 (kappa=0.36, *P*=0.000) (Table 2). For patients with COPD, in terms of the agreement of group assignment, either no substantial or almost perfect agreement (kappa>0.6) was found between the cut-point CAT score ⩾10 and each mMRC cut-point.

Concordance was defined as the COPD group being classified based on the CAT score that was consistent with that defined by the mMRC scale. Conversely, discordance was defined as the COPD group being classified based on the CAT score, which was inconsistent with that defined by the mMRC scale. Table 1 shows that 38.2% (289/757) of the enrolled participants had a discordance in group assignment. Compared with the concordant group, the discordant group had a lower mean CAT score (7.9±5.1 versus 12.2±7.8, *P*=0.000), less severe airway obstruction (FEV_1_/FVC of 56.1±8.9 versus FEV_1_/FVC of 54.2±10.2, *P*=0.009) and less severe airflow limitation (FEV_1_% predicted of 56.9±20.7 versus FEV_1_% predicted of 54.0±22.1, *P*=0.072).

The discordance group was divided into two subgroups: CAT⩾10/mMRC 0–1 and CAT<10/mMRC⩾2. In all, 28.7% (83/289) and 71.3% (206/289) of participants with discordant group assignment had CAT⩾10/mMRC 0–1 and CAT<10/mMRC⩾2, respectively (Table 3). Compared with the CAT<10/mMRC⩾2 subgroup, CAT⩾10/mMRC 0–1 subgroup had a higher percentage of presence of wheezing.

Table 4 shows that a moderate correlation existed between CAT and mMRC in the study population (*r*=0.480, *P*=0.000*). The regression model was CAT=3.724+3.685×mMRC (*R*^2^=0.230, F=225.440, *P*=0.000). More-symptom groups (e.g., groups B, D and B+D) had moderate correlations between these questionnaires whether classified by CAT or mMRC, whereas less symptom groups (e.g., groups A, C, A+C) had only weak-to-modest correlations except for the group C classified by mMRC. Moreover, the combined high-risk group (group C+D) had a moderate correlation between these questionnaires, whereas the combined low-risk group (group A+B) only had a modest correlation.

**Figure 1:**
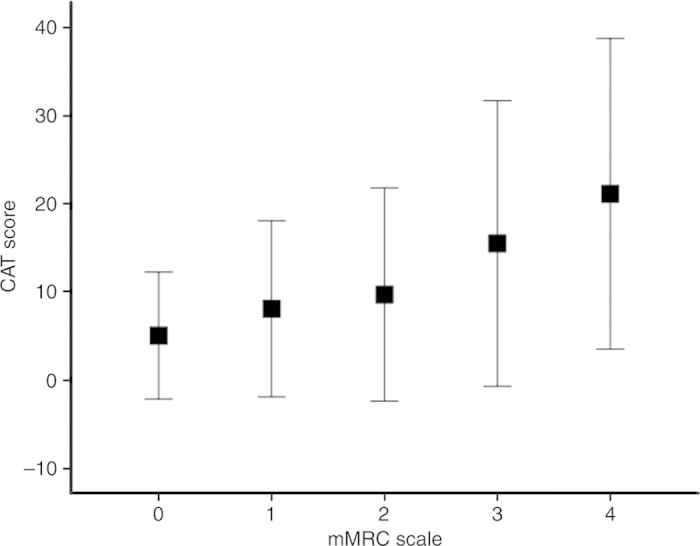
The relationship between the CAT score and mMRC scale for the study COPD population. Data were presented as mean±2 s.d. *P*=0.000 for the one-way ANOVA of the association between the CAT score and mMRC scale.

Figure 1 illustrates that a significant positive relationship existed between the CAT score and mMRC scale (*P*=0.000). The mean CAT score ⩾10 occurred when the mMRC scale was ⩾3. Notably, a significant proportion of COPD patients with each scale of mMRC had health status impairment (CAT score ⩾10).

**Figure 2:**
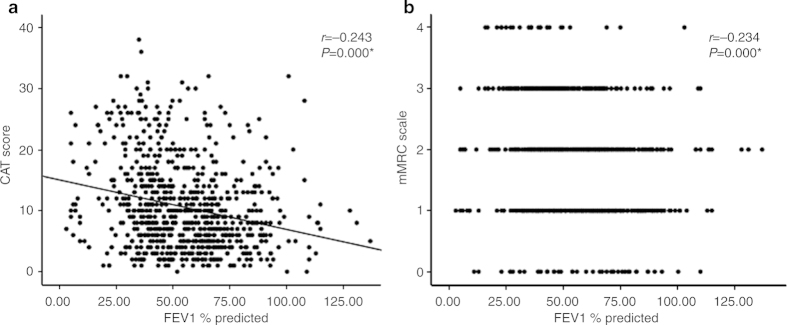
Relationships (**a**) between post-bronchodilator test forced expiratory volume in 1 s % predicted (post-BT FEV_1_% predicted) and the CAT score and (**b**) between post-BT FEV_1_% predicted and the mMRC scale. *P<0.05.

Figure 2 illustrates that the relationships between post-BT FEV_1_% predicted and the CAT score and between post-BT FEV_1_% predicted and the mMRC scale were only modestly negatively correlated.

Compared with those with CAT<10 and mMRC 0–1, COPD patients with CAT⩾10 and mMRC ⩾2 were older in age and had a higher percentage of presence of wheezing, more severe airway obstruction, less FEV_1_ and FVC, more severe airflow limitation, parallel higher mMRC scales and higher CAT scores and more exacerbations, respectively (see Supplementary Tables S1 and S2 in the Supplementary Material).

## Discussion

### Main findings

This study demonstrated that COPD group assignment was not identical when categorised based on the cut-point CAT score ⩾10 and each mMRC cut-point. Furthermore, no substantial or almost perfect agreement presented between the cut-point CAT score ⩾10 and each mMRC cut-point. In all, 38.2% of participants were found to have discordant group assignments together with a lower mean CAT score, less severe airway obstruction and less severe airflow limitation than those with concordant group assignment. In the discordant group, the CAT⩾10/mMRC 0–1 subgroup had a higher percentage of presence of wheezing than the CAT<10/mMRC⩾2 subgroup. Neither perfect nor high correlation existed between CAT and mMRC even though the more-symptom groups and combined high-risk group had better correlations than the less-symptom groups and combined low-risk group, respectively. A modest negative correlation existed between FEV_1_% predicted and CAT score and between FEV_1_% predicted and mMRC scale in parallel with a significant positive relationship existing between the CAT score and mMRC scale. Notably, a significant proportion of COPD patients with each scale of mMRC had health status impairment. COPD patients with more advanced age, wheezing, worse spirometric parameters, including more severe airway obstruction, less FEV_1_ and FVC and more severe airflow limitation, and more exacerbations were associated with a worse health status and respiratory capacity.

### Interpretation of findings in relation to previously published work

Consonant with the present study, several previous studies found that the group assignment of patients with COPD using the symptom assessment methods (CAT or mMRC) proposed by GOLD 2011 was not consistent,^8,11,12^ which may be because the mMRC only assesses disability owing to breathlessness, whereas the CAT has a broader coverage of the impact of COPD on the patient’s daily life. By using these two questionnaires, two previous studies showed that 53.7% (890/1659) and 27.2% (77/283) of enrolled participants had discordant COPD group assignments, respectively;^8,11^ and the present study revealed a discordance rate of 38.2% (289/757). Interestingly, we found that the discordance group had a lower CAT score, less severe airway obstruction and less severe airflow limitation. Furthermore, wheezing was associated with the CAT⩾10/mMRC 0–1 discordant subgroup. Although GOLD 2011 recommended that it is not necessary to use more than one symptom assessment questionnaire, these findings suggest that both simple tools, CAT and mMRC,^5,13^ should be assessed simultaneously for COPD patients with a lower CAT score, less severe airway obstruction and less severe airflow limitation in order to classify patients into optimal COPD groups and optimise COPD management. For COPD patients with inconsistent group assignment by using these two questionnaires, the presence of wheezing may imply not a worse respiratory capacity but a worse health status.

We found that there was a significant positive relationship between the CAT score and mMRC scale even though only a moderate correlation existed between these two questionnaires. We also found that a significant proportion of COPD patients with each scale of mMRC had health status impairment (CAT score ⩾10). These findings were similar with those reported by Jones *et al*.^12^ and shows that, notwithstanding the existence of a positive correlation, the CAT score ⩾10 serving as an equivalence to an mMRC score ⩾2 as recommended by GOLD 2011 may not be applicable to all COPD patients. In other words, COPD patients categorised as having less symptoms using the cut-point mMRC⩾2 (the GOLD 2011 recommendation) may have a worse health status and vice versa.

We observed that a lower mean CAT score for each mMRC scale compared with findings reported in one previous study conducted in a western country.^12^ Although the baseline characteristics of enrolled COPD patients in the present study were similar to several published studies, the distribution of COPD groups was different from one study to another.^7,11,12^ As a result, different geographical and sociocultural environments may have different impacts on symptom assessment and COPD categorisation.

Regarding the cut-points CAT score ⩾10 and mMRC scale ⩾2 that were recommended by GOLD 2011, our study reiterated the findings of several previous studies that reported that these two questionnaires had only moderate agreement in the categorisation of COPD groups.^7,11,12,14^ In contrast, one previous large-scale cohort study found that the cut-points CAT score ⩾10 and mMRC scale ⩾1 were approximately equivalent in determining COPD groups with substantial agreement.^12^ Although the cut-points CAT score ⩾10 and mMRC scale ⩾3 had the best agreement (kappa=0.55, *P*=0.000) in the present study, it was the case that only moderate agreement was observed. Our findings suggest that there was no optimal cut-point for mMRC to correspond to the cut-point CAT score ⩾10, which differed from the results reported in the previously mentioned large-scale study.^12^ This can be explained by the different study designs between these two studies. The large-scale cohort study was conducted in a primary care setting and the number of exacerbations was recorded only in the previous 6 months; also, spirometry was not performed in a standardised way across the study institutes. In contrast, our study design, as mentioned in the paragraph under the heading ‘Strengths and limitations of this study’, was more rigorous and therefore better represented the true distribution of COPD groups and agreement of group assignments between the CAT score and the mMRC scale of COPD patients.

Although COPD patients with worse spirometric parameters were associated with a worse health status (CAT⩾10) and respiratory capacity (mMRC⩾2), along with our findings, several studies found that respiratory disease questionnaires, including St George’s Respiratory Questionnaire total scores, CAT scores and mMRC scales, had only a weak negative correlation with FEV_1_% predicted.^15,16^ This indicates that, with any given pulmonary function reserve, COPD patients may range from having relatively well-preserved to very poor health status and from no respiratory disability to almost complete respiratory incapacity.

One previous global trial found that 13.6% of moderate-to-very-severe COPD patients were frequent exacerbators who were closely associated with exacerbation-related hospitalisations and poorer survival.^17^ We found that 13.6% of mild-to-very-severe COPD patients had frequent exacerbation. Furthermore, more exacerbations in the previous year were associated with a worse health status (CAT⩾10) and respiratory capacity (mMRC⩾2). This indicates that, compared with non-frequent exacerbators, frequent exacerbators had a worse health status and respiratory capacity, which may explain why frequent exacerbators tended to have exacerbation-related hospitalisations and poorer survival.^17^

One previous study indicated that only 2.6% (6/230) of non-smoker COPD patients and 1.9% (7/375) of smoker COPD patients had a history of wheezing at the outpatient clinics.^18^ We found that 44.8% of stable COPD patients had wheezing and were associated with a worse health status (CAT⩾10) and respiratory capacity (mMRC⩾2), compared with those without wheezing. However, the implications remain unclear regarding the association of wheezing and important clinical outcomes (such as the response to inhaled corticosteroids or long-acting β_2_-agonist), risks of exacerbation and hospitalisation and mortality in stable COPD patients. Further research should thus explore the clinical relevance of wheezing in stable COPD patients.

Unlike the Understanding Potential Long-term Impacts on Function with Tiotropium study and the report by Zhang *et al*.^18^ showing a bronchodilator reversibility in 53.9% and 8.4% of COPD patients, respectively, we found that 30.5% of COPD patients exhibited airway reversibility.^18,19^ Along with our findings, other evidence suggests that reversibility results cannot predict a COPD patient’s quality of life, respiratory capacity or long-term response to maintenance bronchodilator treatment.^20,21^

Similar to the results in a cross-sectional study conducted in Europe,^15^ we found that there were no associations between any COPD-related co-morbidity and health status and between any COPD-related co-morbidity and respiratory capacity. These findings indicate that the quality of life and respiratory capacity of COPD patients were not affected by any COPD-related co-morbidity. However, COPD patients with simultaneous ⩾3 co-morbidities would have a higher CAT score.^15^

### Strengths and limitations of this study

This study was implemented by qualified pulmonologists who were actively involved in COPD management in a referral hospital setting, and in order to comply with the GOLD 2011 recommendation, the number of exacerbations was recorded in the previous 12 months; we also performed spirometry according to the American Thoracic Society statement in all study institutes. We believe that our study design was rigorous and therefore represented the true distribution of COPD groups and agreement of group assignments between the CAT score and the mMRC scale of COPD patients.

There were several limitations in our study. First, instead of recording all COPD-related co-morbidities, we only kept a record of the co-morbidities of interest, including cardiovascular diseases, chronic lung diseases and lung cancer. As a result, the effect of COPD-related co-morbidities on health status and respiratory capacity could not be evaluated comprehensively. Second, the participants were not sampled randomly. COPD patients with worse health status and respiratory capacity (e.g., CAT score ⩾30 and mMRC scale=4) had less will to participate in the study. Hence, underestimations of the overall CAT scores and mMRC scales, the proportion of more-symptom group and the proportion of concordant group assignment may exist. Third, as few as 7.9% (60/757) of participants were non-smoker COPD patients in the present study. Therefore, our findings may not be applicable to patients with COPD who are associated with risk factors other than cigarette smoking.

### Implications for future research, policy and practice

In contrast to the GOLD 2011 recommendation that either the CAT score or mMRC scale can be the symptom assessment questionnaire, we have shown evidence that both the CAT score and mMRC scale should be evaluated simultaneously for COPD patients with a lower CAT score, less severe airway obstruction and less severe airflow limitation. Although GOLD 2011 recommended that CAT is preferred as it provides a more comprehensive assessment of the symptomatic impact on patients with COPD, how to categorise COPD patients with inconsistent group assignment based on these two respiratory questionnaires to optimise COPD management as well as how to choose more suitable questionnaires for special types of COPD patients need to be validated further in future studies.

### Conclusions

In contrast to the GOLD 2011 recommendation, COPD patients with a lower CAT score, less severe airway obstruction and less severe airflow limitation were associated with discordant group assignments and should evaluate their symptoms with both CAT and mMRC simultaneously. We did not find any optimal cut-point for mMRC to correspond to the cut-point CAT score ⩾10. The GOLD committee should redefine the applications of CAT and mMRC in the management of COPD.

## The TOLD study group

The principal investigators of the TOLD study group include Ying-Huang Tsai^8^, Chi-Wei Tao^9^, Shih-Lung Cheng^10^, Chao-Hsien Lee^11^, Ping-Hung Kuo^12^, Yao-Kuang Wu^13^, Ning-Hung Chen^14^, Wu-Huei Hsu^15^, Jeng-Yuan Hsu^16^, Ming-Shian Lin^17^, Chin-Chou Wang^18^ and Yu-Feng Wei^19^

^8^Chang Gung Memorial Hospital, Chiayi, Taiwan;

^9^Cheng-Hsin General Hospital, Taipei, Taiwan;

^10^Far Eastern Memorial Hospital, Taipei, Taiwan;

^11^Mackay Memorial Hospital, Taipei, Taiwan;

^12^National Taiwan University Hospital, Taipei, Taiwan;

^13^Taipei Tzu Chi Hospital, Taipei, Taiwan;

^14^Chang Gung Memorial Hospital, Linkou, Taiwan;

^15^China Medical University Hospital, Taichung, Taiwan;

^16^Taichung Veterans General Hospital, Taichung, Taiwan;

^17^Chia-Yi Christian Hospital, Chiayi, Taiwan;

^18^Chang Gung Memorial Hospital, Kaohsiung, Taiwan;

^19^E-DA Hospital, Kaohsiung, Taiwan.

## Figures and Tables

**Table 1:** Baseline characteristics of the enrolled participants (*n*=757)

	*Concordance (*n*=468)*	*Discordance (*n*=289)*	*Total (*n*=757)*	P*value*
*Age*^a^	72.1±9.3	72.3±9.5	72.2±9.4	0.541
<60	40 (8.5%)	28 (9.7%)	68 (9.0%)	
60–69	138 (29.5%)	78 (27.0%)	216 (28.5%)	
70–79	184 (39.3%)	106 (36.7%)	290 (38.3%)	
⩾80	106 (22.6%)	77 (26.6%)	183 (24.2%)	
				
*Gender*^b^				1.000
Male	450 (96.2%)	278 (96.2%)	728 (96.2%)	
Female	18 (3.8%)	11 (3.8%)	29 (3.8%)	
				
*Smoking*^b^				0.532
Never	37 (7.9%)	23 (8.0%)	60 (7.9%)	
Ex-smoker	267 (57.1%)	176 (60.9%)	443 (58.5%)	
Current smoker	164 (35.0%)	90 (31.1%)	254 (33.6%)	
				
*BMI*^a^	23.1±3.7	23.4±3.8	23.3±3.7	0.308
				
*Wheezing*^b^				0.180
Presence	219 (46.8%)	120 (41.5%)	339 (44.8%)	
Absence	249 (53.2%)	169 (58.5%)	418 (55.2%)	
				
*Spirometry (post-bronchodilator test)*				
FEV_1_/FVC (%)^a^	54.2±10.2	56.1±8.9	54.9±9.7	0.009*
FEV_1_ (L)^a^	1.3±0.5	1.3±0.5	1.3±0.5	0.898
FVC (L)^a^	2.4±0.8	2.3±0.6	2.3±0.7	0.259
FEV_1_% predicted^a^	54.0±22.1	56.9±20.7	55.1±21.6	0.072
				
*Bronchodilator tests*^b^				0.662
Positive	146 (31.2%)	85 (29.4%)	231 (30.5%)	
Negative	322 (68.2%)	204 (70.6%)	526 (69.5%)	
				
*GOLD spirometric classification*				0.064
I	61 (13.0%)	27 (9.3%)	88 (11.6%)	
II	180 (38.5%)	134 (46.4%)	314 (41.5%)	
III	180 (38.5%)	109 (37.7%)	289 (38.2%)	
IV	47 (10%)	19 (6.6%)	66 (8.7%)	
				
*CAT scores*^a^	12.2±7.8	7.9±5.1	10.6±7.2	0.000*
<10	200 (42.7%)	200 (69.2%)	400 (52.8%)	
⩾10	268 (57.3%)	89 (30.8%)	357 (47.2%)	
				
*mMRC*^a^	1.9±1.1	1.9±0.8	1.9±1.0	0.512
0–1	201 (42.9%)	79 (27.3%)	280 (37.0%)	
2–4	267 (57.1%)	210 (72.7%)	477 (63.0%)	
				
*Exacerbation numbers in the previous year*^a^	0.6±1.1	0.6±1.2	0.6±1.1	0.799
0–1	403 (86.1%)	251 (86.9%)	654 (86.4%)	
⩾2	65 (13.9%)	38 (13.1%)	103 (13.6%)	
				
*Inhaled pharmacological therapy*				0.129
None	36 (7.7%)	32 (11.1%)	68 (9.0%)	
LAMA alone	121 (25.9%)	80 (27.7%)	201 (26.6%)	
LABA alone	24 (5.1%)	10 (3.5%)	34 (4.5%)	
LABA+LAMA	31 (6.6%)	18 (6.2%)	49 (6.5%)	
LAMA+ICS	9 (1.9%)	7 (2.4%)	16 (2.1%)	
ICS/LABA	103 (22.0%)	77 (26.6%)	180 (23.8%)	
ICS/LABA+LAMA	144 (30.8%)	65 (22.5%)	209 (27.6%)	
				
Usage of methylxanthines	351 (75.0%)	212 (73.4%)	563 (74.4%)	0.676
				
*Co-morbidities*				
Cardiovascular disease^b^^,^^c^	115 (24.6%)	76 (26.3%)	191 (25.2%)	0.606
Chronic lung disease^b^^,^^d^	42 (9.0%)	23 (8.0%)	65 (8.6%)	0.690
Lung cancer^b^	9 (1.9%)	5 (1.7%)	14 (1.8%)	1.000

Abbreviations: BMI, body mass index; CAT, COPD assessment test; FEV_1_, forced expiratory volume in 1 s; FVC, forced vital capacity; GOLD, Global initiative for Chronic Obstructive Lung Disease; ICS, inhaled corticosteroid; LABA, long-acting β_2_-agonist; LAMA, long-acting muscarinic antagonist; mMRC, modified Medical Research Council dyspnoea scale.

a By independent *t*-test.

b By chi-square test.

c Cardiovascular disease included ischaemic heart disease, heart failure, atrial fibrillation and hypertension.

d Chronic lung disease included previous pulmonary tuberculosis, bronchiectasis and pneumoconiosis.

*P<0.05.

**Table 2:** Proportion of each COPD group (A–D) and agreement when categorised based on the cut-point CAT score ⩾10 and each mMRC cut-point

	*COPD group (%)*				*Kappa value*	P *value*
	*A*	*B*	*C*	*D*		
CAT⩾10	30.6	17.3	22.2	29.6	Reference	
mMRC⩾1	3.2	45.0	2.4	49.4	0.36	0.000*
mMRC⩾2	22.2	26.0	15.9	35.9	0.49	0.000*
mMRC⩾3	41.6	6.6	34.6	17.2	0.55	0.000*
mMRC=4	48.0	0.3	47.8	0.4	0.40	0.000*

Abbreviations: CAT, COPD assessment test; COPD, chronic obstructive pulmonary disease; mMRC, modified Medical Research Council dyspnoea scale. *P<0.05.

**Table 3:** Detailed characteristics in COPD patients with discordant group assignments when divided into CAT⩾10/mMRC 0–1 and CAT<10/mMRC⩾2 subgroups

	*CAT⩾10/mMRC 0–1 (*n*=83)*	*CAT<10/mMRC⩾2 (*n*=206)*	P *value*
Age^a^	71.7±10.2	72.5±9.2	0.485
			
*Gender*^b^			0.362
Male	78 (94.0%)	200 (97.1%)	
Female	5 (6.0%)	6 (2.9%)	
			
*Smoking*^b^			0.132
Never	8 (9.6%)	15 (7.3%)	
Ex-smoker	43 (51.8%)	133 (64.6%)	
Current smoker	32 (38.6%)	58 (28.2%)	
			
BMI^a^	23.5±3.5	23.4±3.9	0.846
			
*Wheezing*^b^			0.008*
Presence	45 (54.2%)	75 (36.4%)	
Absence	38 (45.8%)	131 (63.6%)	
			
*Spirometry (post- bronchodilator test)*			
FEV_1_/FVC (%)^a^	56.4±10.2	55.9±8.3	0.717
FEV_1_ (L)^a^	1.3±0.5	1.3±0.5	0.711
FVC (L)^a^	2.3±0.6	2.3±0.6	0.713
FEV_1_% predicted^a^	55.7±21.9	57.4±20.3	0.535
			
*Bronchodilator tests*^b^			1.000
Positive	24 (28.9%)	61 (29.6%)	
Negative	59 (71.1%)	145 (70.4%)	
			
CAT scores^a^	13.8±4.2	5.5±3.0	0.000*
mMRC^a^	1.0±0.3	2.2±0.5	0.000*
Exacerbation numbers in the previous year^a^	0.7±1.1	0.5±1.2	0.345
			
*Co-morbidities*			
Cardiovascular disease^b^^,^^c^	23 (27.7%)	53 (25.7%)	0.842
Chronic lung disease^b^^,^^d^	4 (4.8%)	19 (9.2%)	0.241
Lung cancer^b^	3 (3.6%)	2 (1.0%)	0.145

Abbreviations: BMI, body mass index; CAT, COPD assessment test; COPD, chronic obstructive pulmonary disease; FEV_1_, forced expiratory volume in 1 s; FVC, forced vital capacity; mMRC, modified Medical Research Council dyspnoea scale.

a By independent *t*-test.

b By chi-square test.

c Cardiovascular disease included ischaemic heart disease, heart failure, atrial fibrillation and hypertension.

d Chronic lung disease included previous pulmonary tuberculosis, bronchiectasis and pneumoconiosis.

*P<0.05.

**Table 4:** Correlations between CAT and mMRC in each COPD group (A–D) and combined COPD groups when categorised based on GOLD 2011

	*Assignment by CAT*		*Assignment by mMRC*	
	*Number (%)*	*Pearson’s correlation*	*Number (%)*	*Pearson’s correlation*
*Individual COPD group*				
A	232 (30.6)	*r*=0.010 *P*=0.879	168 (22.2)	*r*=0.189 *P*=0.014*
B	133 (17.6)	*r*=0.401 *P*=0.000*	197 (26.0)	*r*=0.431 *P*=0.000*
C	168 (22.2)	*r*=−0.060 *P*=0.437	120 (15.9)	*r*=0.465 *P*=0.000*
D	224 (29.6)	*r*=0.462 *P*=0.000*	272 (35.9)	*r*=0.415 *P*=0.000*
				
*Combined COPD group*				
A+C	400 (52.8)	*r*=−0.018 *P*=0.713	288 (38.0)	*r*=0.357 *P*=0.000*
B+D	357 (47.2)	*r*=0.460 *P*=0.000*	469 (62.0)	*r*=0.446 *P*=0.000*
A+B	365 (48.2), *r*=0.391, *P*=0.000*			
C+D	392 (51.8), *r*=0.480, *P*=0.000*			
	
All	757 (100.0), *r*=0.480, *P*=0.000*			

Abbreviations: CAT, COPD assessment test; COPD, chronic obstructive pulmonary disease; GOLD, Global initiative for Chronic Obstructive Lung Disease; mMRC, modified Medical Research Council dyspnoea scale. *P<0.05.
