# Supplementary material for: Features of COPD patients by comparing CAT with mMRC: a retrospective, cross-sectional study
Source: NPJ Prim Care Respir Med. 2015 Nov 5;25:15063–. doi: 10.1038/npjpcrm.2015.63 (PMC4633725; doi:10.1038/npjpcrm.2015.63)
Supplement: Supplementary Information [file npjpcrm201563-s1.doc]

Table S1. The detailed characteristics between COPD patients with poor health status (CAT≧10) and those with preserved health status (CAT＜10)

|  | CAT≧10 (n=357) | CAT＜10 (n=400) | Total  (n=757) | p value |
| --- | --- | --- | --- | --- |
| Age^†^ | 72.9±9.5 | 71.5±9.3 | 72.2±9.4 | 0.036* |
| Gender^‡^ |  |  |  | 0.755 |
| Male | 342 (95.8%) | 386 (96.5%) | 728 (96.2%) |  |
| Female | 15 (4.2%) | 14 (3.5%) | 29 (3.8%) |  |
| Smoking^‡^ |  |  |  | 0.331 |
| Never | 33 (9.2%) | 27 (6.8%) | 60 (7.9%) |  |
| Ex- smoker | 201 (56.3%) | 242 (60.5%) | 443 (58.5%) |  |
| Current smoker | 123 (34.5%) | 131 (32.8%) | 254 (33.6%) |  |
| BMI^†^ | 23.0±3.9 | 23.5±3.6 | 23.3±3.7 | 0.080 |
| Wheezing^‡^ |  |  |  | 0.000* |
| Presence | 201 (56.3%) | 138 (34.5%) | 339 (44.8%) |  |
| Absence | 156 (43.7%) | 262 (65.5%) | 418 (55.2%) |  |
| Spirometry (Post- bronchodilator test) | | | |  |
| FEV1/ FVC (%)^†^ | 52.8±10.4 | 56.8±8.7 | 54.9±9.7 | 0.000* |
| FEV1 (L) ^†^ | 1.2±0.5 | 1.4±0.5 | 1.3±0.5 | 0.000* |
| FVC (L) ^†^ | 2.2±0.7 | 2.4±0.7 | 2.3±0.7 | 0.000* |
| FEV1 % predicted^†^ | 50.1±21.1 | 59.6±21.1 | 55.1±21.6 | 0.000* |
| Bronchodilator tests^‡^ |  |  |  | 0.929 |
| Positive | 110 (30.8%) | 121 (30.3%) | 231 (30.5%) |  |
| Negative | 247 (69.2%) | 279 (69.8%) | 526 (69.5%) |  |
| CAT scores^†^ | 16.6±5.9 | 5.2±2.4 | 10.6±7.2 | 0.000* |
| mMRC^†^ | 2.2±0.9 | 1.5±0.8 | 1.9±1.0 | 0.000* |
| Exacerbation numbers in the previous year^†^ | 0.8±1.2 | 0.4±1.0 | 0.6±1.1 | 0.000* |
| Co-morbidities |  |  |  |  |
| Cardiovascular disease^‡＃^ | 86 (24.1%) | 105 (26.3%) | 191 (25.2%) | 0.549 |
| Chronic lung disease^‡※^ | 24 (6.7%) | 41 (10.3%) | 65 (8.6%) | 0.110 |
| Lung cancer^‡^ | 9 (2.5%) | 5 (1.3%) | 14 (1.8%) | 0.305 |

*: *p*<0.05

Abbreviations: see Table 1.

^†^by independent t test.

^‡^by chi-square test.

^＃^Cardiovascular disease included ischemic heart disease, heart failure, atrial fibrillation and hypertension.

^※^Chronic lung disease included previous pulmonary tuberculosis, bronchiectasis and pneumoconiosis.

Table S2. The detailed characteristics between COPD patients with more respiratory disability (mMRC≧2) and those with less respiratory disability (mMRC 0-1)

|  | mMRC≧2 (n=477) | mMRC 0-1 (n=280) | Total  (n=757) | p value |
| --- | --- | --- | --- | --- |
| Age^†^ | 73.1±9.2 | 70.6±9.6 | 72.2±9.4 | 0.000* |
| Gender^‡^ |  |  |  | 0.487 |
| Male | 461 (96.6%) | 267 (95.4%) | 728 (96.2%) |  |
| Female | 16 (3.4%) | 13 (4.6%) | 29 (3.8%) |  |
| Smoking^‡^ |  |  |  | 0.205 |
| Never | 40 (8.4%) | 20 (7.1%) | 60 (7.9%) |  |
| Ex- smoker | 288 (60.4%) | 155 (55.4%) | 443 (58.5%) |  |
| Current smoker | 149 (31.2%) | 105 (37.5%) | 254 (33.6%) |  |
| BMI^†^ | 23.1±3.9 | 23.5±3.4 | 23.3±3.7 | 0.139 |
| Wheezing^‡^ |  |  |  | 0.024* |
| Presence | 229 (48.0%) | 110 (39.3%) | 339 (44.8%) |  |
| Absence | 248 (52.0%) | 170 (60.7%) | 418 (55.2%) |  |
| Spirometry (Post- bronchodilator test) | | | |  |
| FEV1/ FVC (%)^†^ | 53.6±9.7 | 57.1±9.5 | 54.9±9.7 | 0.000* |
| FEV1 (L) ^†^ | 1.2±0.5 | 1.5±0.5 | 1.3±0.5 | 0.000* |
| FVC (L) ^†^ | 2.2±0.7 | 2.5±0.7 | 2.3±0.7 | 0.000* |
| FEV1 % predicted^†^ | 52.4±21.0 | 59.8±21.8 | 55.1±21.6 | 0.000* |
| Bronchodilator tests^‡^ |  |  |  | 0.799 |
| Positive | 144 (30.2%) | 87 (31.1%) | 231 (30.5%) |  |
| Negative | 333 (69.8%) | 193 (68.9%) | 526 (69.5%) |  |
| CAT scores^†^ | 12.3±7.8 | 7.6±4.9 | 10.6±7.2 | 0.000* |
| mMRC^†^ | 2.4±0.6 | 0.9±0.4 | 1.9±1.0 | 0.000* |
| Exacerbation numbers in the previous year^†^ | 0.7±1.3 | 0.4±0.8 | 0.6±1.1 | 0.001* |
| Co-morbidities |  |  |  |  |
| Cardiovascular disease^‡＃^ | 117 (24.5%) | 74 (26.4%) | 191 (25.2%) | 0.621 |
| Chronic lung disease^‡※^ | 39 (8.2%) | 26 (9.3%) | 65 (8.6%) | 0.695 |
| Lung cancer^‡^ | 8 (1.7%) | 6 (2.1%) | 14 (1.8%) | 0.857 |

*: *p*<0.05

Abbreviations: see Table 1.

^†^by independent t test.

^‡^by chi-square test.

^＃^Cardiovascular disease included ischemic heart disease, heart failure, atrial fibrillation and hypertension.

^※^Chronic lung disease included previous pulmonary tuberculosis, bronchiectasis and pneumoconiosis.
